# Treatment standards for direct oral anticoagulants in patients with acute ischemic stroke and non-valvular atrial fibrillation: A survey among German stroke units

**DOI:** 10.1371/journal.pone.0264122

**Published:** 2022-02-17

**Authors:** Christian Fastner, Kristina Szabo, Melina Samartzi, Mathieu Kruska, Ibrahim Akin, Michael Platten, Stefan Baumann, Angelika Alonso

**Affiliations:** 1 Department of Cardiology, Angiology, Haemostaseology and Medical Intensive Care, University Medical Centre Mannheim, Medical Faculty Mannheim, Heidelberg University, European Center for AngioScience (ECAS), and German Center for Cardiovascular Research (DZHK) partner site Heidelberg/Mannheim, Mannheim, Germany; 2 Department of Neurology, University Medical Centre Mannheim, Mannheim Center for Translational Neurosciences, Medical Faculty Mannheim, Heidelberg University, Mannheim, Germany; Leiden University Medical Center, NETHERLANDS

## Abstract

**Background:**

Acute ischemic stroke (AIS) in patients with non-valvular atrial fibrillation (AF) despite oral anticoagulation (OAC) is a complex and insufficiently investigated setting. Potential strategies range from maintaining the current OAC to changing the substance class. We have queried the specific treatment standards on German stroke units (SUs).

**Methods:**

By means of a standardized online questionnaire via SurveyMonkey™ (San Mateo, CA, USA), all clinical heads of German SUs were asked about their treatment standards in the following clinical situations: first AIS of an OAC-naïve AF patient, AF patient with AIS despite administration of a vitamin K antagonist (VKA), AF patient with AIS despite administration of direct OAC (DOAC). In addition, the performance of specific coagulation tests in AF patients with AIS despite OAC was queried.

**Results:**

160 (48%) clinical heads of German SU responded. Data from pivotal trials (84%), own experience with substances (71%), and side-effect profiles (66%) determine the initial DOAC prescription. In case of an AIS despite OAC, 83 and 18% would switch from VKA to DOAC under certain conditions and always, respectively. Half of respondents would switch from DOAC to VKA under certain conditions, while the other half would decline. 96% would switch to an alternative DOAC. The vast majority of those who made preconditions considered concomitant diseases (92, 90, and 81%, respectively). Few would consider infarct pattern (<35%). 61% perform initial coagulation tests (only one-third substance-specific assessments); however, the majority do not use these to make further decisions.

**Conclusions:**

In the setting of an OAC-naïve AF patient with AIS, established pivotal data are most respected. In the unclear setting of an AIS despite OAC, most respondents consider concomitant diseases and give preference to switching to a (different) DOAC.

## Introduction

Atrial fibrillation (AF) is a major cause of acute ischemic stroke (AIS), and the risk of AIS attributable to AF steeply increases with age [1]. Of more than 180,000 patients admitted to stroke units (SU) in Germany in 2012, 25.8% had AF [2]. Oral anticoagulants (OAC) effectively reduce the risk of AIS in patients with AF [3, 4]. Over the last decade, prescriptions of vitamin K antagonists (VKA) have decreased in favor of direct oral anticoagulants (DOAC), which have shown to be an equal or superior alternative with favorable risk-benefit profile [5]. Accordingly, the 2019 European Stroke Organization (ESO) guidelines recommend DOAC over VKA for secondary prevention in patients with non-valvular AF and previous AIS or transient ischemic attack [3].

While there is broad consensus both from neurological and cardiological perspective on the initiation of OAC in treatment-naive patients with AF and AIS [3, 4], it remains unclear whether to change the type of OAC in case of AIS despite OAC treatment. So far, randomized trials addressing this issue are lacking. In order to evaluate current treatment standards regarding OAC in patients with AIS and non-valvular AF, we conducted an online survey in German SU.

## Materials and methods

At the target date of February 2021, 332 SU were certified according to the guidelines of the German Stroke Society (*Deutsche Schlaganfallgesellschaft*, *DSG*). All clinical heads of neurological departments with certified SU were contacted postally and asked to forward the invitation to participate in a standardized online survey via QS code scan to the clinical head of the respective SU. The DSG supported the project. The online survey was performed using the commercially available SurveyMonkey™ (San Mateo, CA, USA) software. Participation was anonymous.

The survey consisted of 13 questions. The first 2 questions explored the characteristics of the participating SU and enquired the categorization as regional or trans-regional SU as well as the number of certified monitoring beds. Trans-regional SUs require the presence of complementary disciplines with 24/7 availability of diagnostic procedures as well as a higher nurse-patient ratio than regional SUs. Questions #3 und #4 addressed the estimated number of patients per year with AIS and non-valvular AF and the estimated number of patients per year with AIS and AF despite treatment with OAC. Questions #5 to #11 covered prescription standards and preferences for OAC with a focus on the practice of switching OAC. Questions #12 and #13 explored the performance of specific coagulation tests in patients on DOAC medication. For these questions, more than one answer could be given by the same respondent. The survey was open from March to May 2021.

The results were exported from SurveyMonkey™ and analyzed by Statistical Package for the Social Sciences (SPSS^®^) version 25 from IBM^®^ (Armonk, NY, USA). Frequencies are given as absolute numbers (n), range (where appropriate) and percentage (%). All response frequencies were compared between the groups regional versus trans-regional SUs using the chi-squared test or the Fisher’s exact test in case of frequencies <5. The comparison of the type of the SUs (i.e., regional versus trans-regional) included in this study with the total of German SUs was performed using the Z-transform. The use of DOAC-specific coagulation tests in regional versus trans-regional SU was investigated by the Fisher’s exact test with Freeman-Halton extension.

## Results

**Fig 1:**
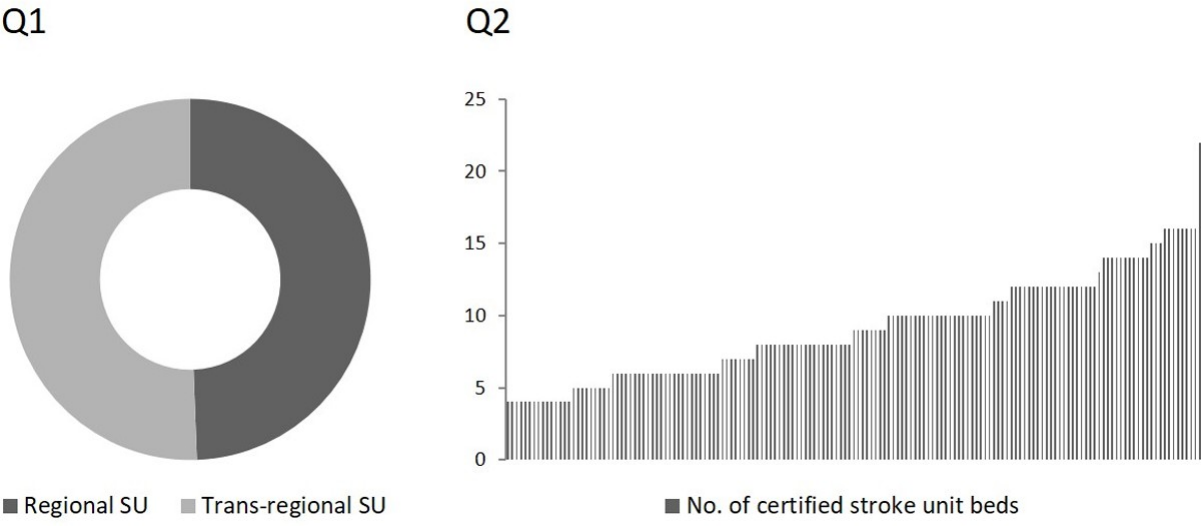
*Question (Q) 1*: Please indicate if our stroke unit is a regional or a trans-regional stroke unit (SU).

In total, 160 heads of SUs responded to our online survey, resulting in a participation rate of 48.2%. Regional SUs were represented by 79 replies (49.4%) whereas 81 replies (50.6%) were ascribed to trans-regional SUs. The number of certified stroke beds per SU ranged from 4 to 22, with a median of 9 (*Fig 1*). There was no significant difference in the size distribution of these SUs compared with the total of German SUs (p = 0.2937).

*Question 2*: Please report the number of certified stroke beds in your stroke unit.

Estimations of the number of patients with AIS and AF per SU varied considerably, ranging from 40 to 1,000 patients/year (median 200/year, R = 960) and amounting to a total estimate of 39,032 patients/year with AIS and AF. The estimated number of patients with AIS and AF under medication with DOAC added up to 13,647 patients/year ranging from 5 to 600 patients/year (median 50/year, R = 595). According to the estimates, about every third patient (35.0%) with an AIS and AF is under treatment with DOAC at the time of the event.

### DOAC first prescription

The decision for a specific *DOAC when first prescribed* was mainly based on published data from pivotal trials (n = 134, 83.8%), own experience (n = 114, 71.3%), and side-effect profile (n = 105, 65.6%). The majority also responded that published real-world data were considered (n = 94, 58.8%) whereas less than half of the responders rated drug interactions (n = 71, 44.4%) and dosing interval (n = 70, 43.8%) as decisive. Among 52 free-text responses, 26 (16.3%) participants named availability/costs of a specific antidot, and 15 responders (9.4%) quoted renal function.

### AIS despite OAC

**Fig 2:**
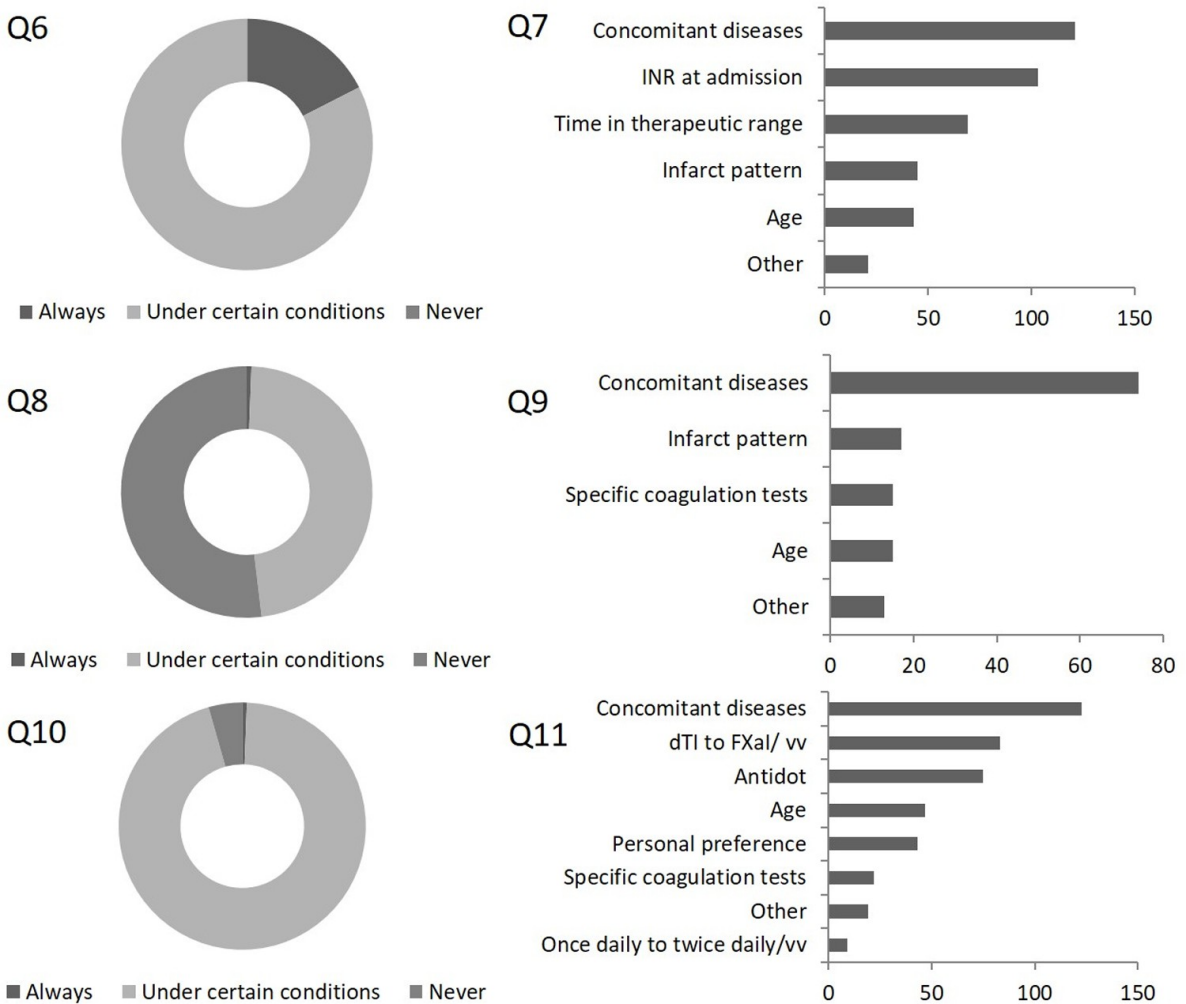
*Question (Q) 6*: Do you switch anticoagulation from vitamin K antagonists (VKAs) to direct oral anticoagulants (DOACs) in patients who suffer an acute ischemic stroke (AIS) under VKA treatment?

In patients with *AIS and AF despite treatment with VKA*, the majority of participants (n = 132, 82.5%) would consider a switch to a DOAC under certain conditions whereas 28 responders (17.5%) would switch to DOACs in any case. Concomitant diseases such as renal insufficiency were named as decisive in 121/132 (91.7%) of cases, followed by coagulation parameters (international normalized ratio (INR) at admission, n = 103, 78.0%; time in therapeutic range (TTR), n = 69, 43.1%). Infarct pattern (n = 45, 34.1%) and age (n = 43, 32.6%) were claimed less frequently. In reverse case, only 76 respondents (47.5%) would consider switching from a DOAC to a VKA under certain conditions if an *AIS occurred despite DOAC treatment* whereas 83 (51.9%) stated that they would not switch to a VKA in any case. Most frequently named reasons for switching to a VKA were concomitant diseases such as renal insufficiency (n = 68, 89.5%). Incident-related factors such as infarct pattern or specific coagulation tests at admission were chosen by 17/76 (22.4%) and 15/76 (19.7%) participants, respectively. Age was respected by 15/76 (19.7%) participants. The vast majority (n = 152, 95%) would consider a switch to an alternative DOAC in patients suffering an AIS despite DOAC treatment whereas only 7 participants (4.4%) responded that they would not approve a switch from one to another DOAC. Again, concomitant diseases were most frequently named as treatment-decisive by 123/152 (80.9%) participants. 83/152 (54.6%) participants would consider switching the mechanism of action, i.e., switching from a direct thrombin inhibitor to a direct factor Xa inhibitor and vice versa. Nearly half of the participants (n = 76) claimed that availability/costs of a specific antidot would influence their decision. Age and personal preferences for a specific DOAC were named by 47/152 (30.9%) and 43/152 (28.3%) respondents, respectively, whereas specific coagulation tests at admission would only be considered by 22/152 (14.5%) participants. Among 28 free-text responses, 9 participants would prefer a switch of dosing intervals, either from twice daily to once daily or vice versa (*Fig 2*). Preferences in switching OAC did not differ between stroke physicians in regional versus trans-regional SUs in the presented settings (VKA to DOAC, p = 0.58; DOAC to VKA, p = 0.39; DOAC to alternative DOAC, p = 0.87; S1 Table).

*Question 7*: If you chose „under certain conditions“: which factors do you consider as treatment-decisive (multiple selection possible)?

*Question 8*: Do you switch anticoagulation from DOACs to VKAs in patients who suffer an AIS under DOAC treatment?

*Question 9*: If you chose „under certain conditions“: which factors do you consider as treatment-decisive (multiple selection possible)?

*Question 10*: Do you switch anticoagulation from one DOAC to another DOAC in patients who suffer an AIS under DOAC treatment?

*Question 11*: If you chose „under certain conditions“: which factors do you consider as treatment-decisive (multiple selection possible)?

INR, international normalized ratio; dTI, direct thrombin inhibitor; FXaI, factor Xa inhibitor; vv, vice versa

**Fig 3:**
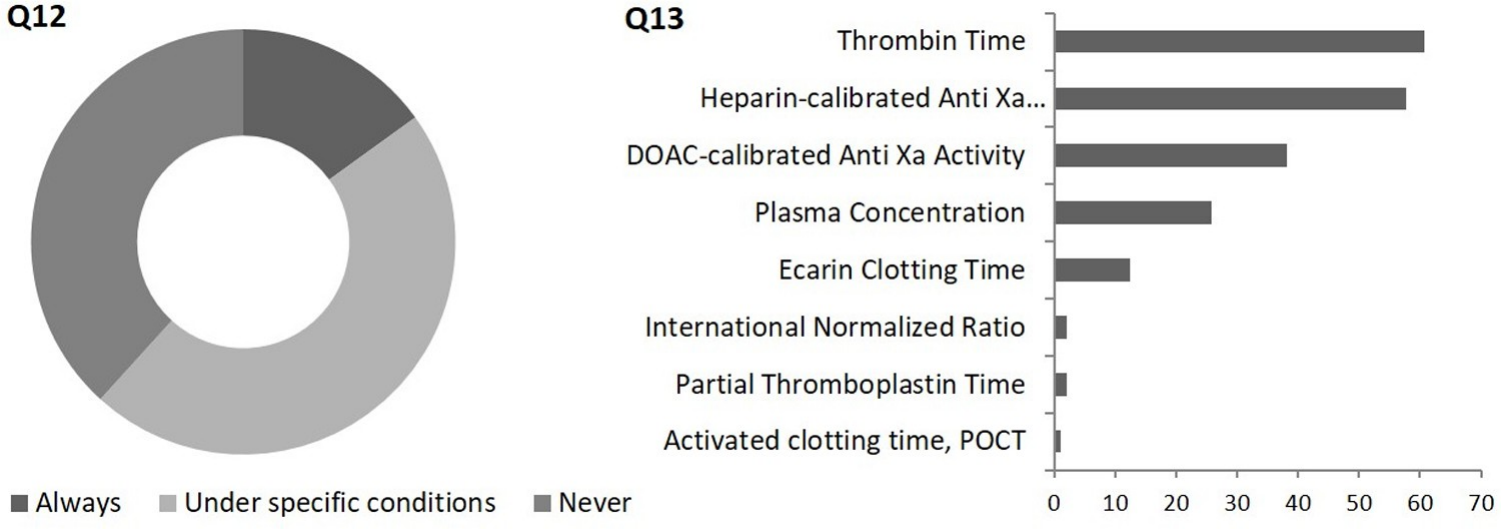
*Question (Q) 12* (n = 160): Do you perform specific coagulation tests in patients with an acute ischemic stroke and non-valvular atrial fibrillation under treatment with direct oral anticoagulants?

With 60.6%, the rate of respondents who state to perform coagulation tests at admission in patients with AIS despite DOAC treatment (77 under certain conditions and 20 always) overweighs the number of participants who use these results as decision guidance for further OAC by far. Assessment of thrombin time (60.8%) and heparin-calibrated anti Xa activity (57.7%) are most frequently performed whereas substance-specific assessments such as DOAC-calibrated anti Xa activity and DOAC plasma concentration were only named by 38.1% and 25.8% of participants, respectively (*Fig 3*). Coagulation tests were performed significantly more frequently in trans-regional SUs (p = 0.004).

*Question 13* (n = 97): If you chose „yes”or „under specific conditions“: Which parameters do you analyse?

POCT, point of care testing

## Discussion

The occurrence of an AIS in an AF patient on DOAC treatment presents practitioners with a great challenge to select the optimal preventive strategy, as DOACs are considered the gold standard in the primary prevention of an AIS in AF patients [4]. Scientific data on this situation are scarce. Recently, Seiffge et al. addressed this issue in a meta-analysis [6]. Although they found an increased rate of recurrent AIS in this patient group, they could not identify any beneficial treatment strategy. The lack of evidence as well as the complexity of the situation is reflected in a wide diversity of strategies, which was extracted from the survey on the current clinical reality at German SUs.

When first admitting a DOAC to an AF patient after AIS, the vast majority of practitioners rely on data from the pivotal trials (84%) [7–10]. This approach is supported by findings confirming the success in AIS prevention and the low adverse event rates of DOACs in secondary prevention, consistent with the pivotal trials [11]. In addition to the practitioners’ own experiences (71%), the side-effect profile was the third most frequently mentioned (66%). This is not surprising, as differences in the side-effect profiles, especially regarding increased gastrointestinal bleeding rates with rivaroxaban versus phenprocoumon, were published from German clinical practice [12]. Though clinically relevant, not even half of practitioners based their decision on drug-drug interactions (44%). Unfortunately, this topic still receives too little attention [13, 14].

The estimated number of AIS in AF patients under DOAC varied among SUs, with a median of about one third of cases, which is overall consistent with data from other groups [6]. Thus, this is a quite common scenario in routine daily stroke care. Previous work identified nonadherence or prescribing errors as causative factors [15, 16]. In addition, risk factors not included in the CHA_2_DS_2_-VASc score or those that do cause non-cardioembolic AIS, play an important role for recurrent event in AF patients on DOAC therapy [6, 16, 17]. Stretz and colleagues postulated four categories of causes for an AIS despite OAC, which include the aforementioned ones: (1) failure of previous OAC despite adequate prescription and intake, (2) prescription or intake error, (3) macrovascular or microvascular cause of AIS, or (4) non-AF-related cardioembolism. Thus, the previously prescribed OAC should not be held solely responsible for the occurrence of an AIS.

When querying strategies for prophylaxis after an AIS in an AF patient despite DOAC treatment, the following major findings were evident: *1) Practitioners are more likely to switch from a VKA to a DOAC than vice versa (90 vs*. *48%)*. *2) Practitioners are very likely to switch between different DOACs in this situation (96%)*. *3) Practitioners base their decision to a greater extent on individual patient factors than on ischemic incident-related factors*. Nevertheless, it is interesting to note that less than a quarter of respondents switch from a VKA to a DOAC in any case, as numerous large studies proofed beneficial benefit-adverse effect profile compared with VKAs for the latter substances [5, 12]. In this regard, many practitioners base their decision on the INR value on admission and the history of TTR. A TTR of >70% is associated with adequate AIS prevention, which is probably equivalent to (most) DOACs [4, 18]. However, bleeding complications are lower with DOACs in general, so the 2019 ESO as well as the 2020 European Society of Cardiology guidelines (class I, level A) preferentially recommend the use of DOACs in patients with nonvalvular AF [3, 4]. In addition, AIS occurring under DOAC treatment were less severe and associated with a lower grade of disability at three months compared to VKA, and stroke volume was smaller [19, 20]. A majority cited the patient’s renal function as the basis for decision making, which is the most important patient factor for DOAC dose reduction. There might be a subjective feeling that the required reduced DOAC dosage would be disadvantageous compared with continued VKA therapy. However, a study on this topic showed a significant benefit when patients eligible for DOAC dose reduction received such instead of VKA therapy [21]. Only patients with end-stage renal failure are exempt from the prescription of DOACs in Germany [22], and also in the United States their use in this patient group is controversially discussed [23]. Conversely, almost half of the practitioners switch from a DOAC to a VKA under certain conditions, again very predominantly citing concomitant diseases such as renal insufficiency. This could follow the same reasoning scheme. In a study on 51,000 German health-insured individuals, it was shown that switching from a VKA to a DOAC or vice versa was generally strictly linked to clinical events [24]. After AIS, the adherence rate was very good under both regimens in German clinical practice [25].

Switching between different DOACs has never been investigated in a randomized controlled trial (RCT) with regard to the success in preventing future recurrent acute ischemic neurological events. Seiffge et al. could not find a beneficial effect of DOAC switching in their large meta-analysis, but this could be due to limitations in the external validity of the underlying original data regarding this specific topic [6]. Even after adjusting for pertinent confounders, the recent IAC study found no evidence of a beneficial effect [26]. Rather, AIS despite OAC appears to be an independent risk factor for future events that is poorly controlled by any OAC continuation alone [27]. Only further insights into the underlying pathomechanisms will improve preventive measures in this situation. Nevertheless, DOAC switching is often practiced in clinical reality, which is essentially based on pharmacodynamic and pharmacokinetic considerations. Fifty-two percent of respondents perform DOAC switching to change the mechanism of action (factor Xa to direct thrombin inhibitor or vice versa). However, comparative studies never found differences in effectiveness of preventing AIS among the two classes, which interact at different key points in the same pathway [5, 12], so the individual patient’s response to the respective mechanism might be considered here. Likewise, the dose regimen (once versus twice daily intake), which may play a role in the switch from one to another DOAC, did not yield a difference in effectiveness in preventing thromboembolic events [28]. The presence of a specific antidote was important for almost half of respondents in the context of DOAC switching. The number of stroke specialists using DOAC-specific coagulation tests to test drug adherence and drug effect on admission was higher than expected (64% for both, DOAC-specific calibration for anti-Xa assay or DOAC plasma concentrations). Testing is still not immediately available in many hospitals, and the test procedure is costly [29]. They are, therefore, performed significantly more often in trans-regional SUs.

The prevention of a recurrent AIS despite upfront DOAC remains a multifaceted task that, in addition to optimizing OAC, must take into account competing causes of an AIS and, hereby, extended antithrombotic regimens [30]. However, only about one-third of respondents were interested in the infarct pattern when weighing a substance change, whereas this could reveal non-cardioembolic causes of AIS that are independent of the preceding DOAC or the risk of hemorrhagic transformation of the infarcted area. The presented heterogeneous data from clinical practice in German SUs underline the unmet need to conduct large multicenter RCTs on the effectiveness of DOAC conversion in this setting.

### Limitations

The presented data represent treatment standards of German SUs and not the therapy actually performed in the past. By nature, the response options in a questionnaire are limited, although care was taken to provide a wide range of response options and the possibility of free text entry. Where appropriate, free-text responses were aggregated to present a representative percentage. This survey did not record whether the DOAC administered before admission was correctly dosed according to the medicinal product’s professional information. In addition, the various causes of AIS despite OAC that may have influenced treatment strategies could not be included in detail in the survey. To appreciate the etiologic aspect, we asked for consideration of the infarct pattern. The contextualization presented is subject to discussion and is not derived originally from the answers given.

## Conclusions

Both in the initial setting and in the case of an AIS despite OAC, German stroke experts rely on DOACs. In the case of AIS despite DOAC therapy, switching from one to another DOAC is very common, although there is no scientific evidence for this action. Individual considerations respecting concomitant diseases and patient compliance seem to affect this.

## Supporting information

S1 Table Preferences in switching oral anticoagulation (OAC) in different settings with respect to regional versus trans-regional stroke units (SUs). (DOCX)

## List of abbreviations

AF: atrial fibrillation
AIS: acute ischemic stroke
(D)OAC (: direct) oral anticoagulant/anticoagulation
DSG: Deutsche Schlaganfallgesellschaft (*German Stroke Society*
ESO: European Stroke Organization
INR: international normalized ration
RCT: randomized controlled trial
SU: stroke unit
TTR: time [in] therapeutic range
VKA: vitamin K antagonist
