# Supplementary material for: Treatment standards for direct oral anticoagulants in patients with acute ischemic stroke and non-valvular atrial fibrillation: A survey among German stroke units
Source: PLoS One. 2022 Feb 17;17(2):e0264122. doi: 10.1371/journal.pone.0264122 (PMC8853580; doi:10.1371/journal.pone.0264122)
Supplement: S1 Table — (DOCX) [file pone.0264122.s001.docx]

**Supplementary Table.**

Preferences in switching oral anticoagulation (OAC) in different settings with respect to regional versus trans-regional stroke units (SUs).

*Question (Q) 6:* Do you switch OAC from vitamin K antagonists (VKAs) to direct oral anticoagulants (DOACs) in patients who suffer an acute ischemic stroke (AIS) under VKA treatment?

|  | **Regional SU** | **Trans-regional SU** | p=0.58 |
| --- | --- | --- | --- |
| Always, n (%) | 12 (15.2) | 16 (19.8) |  |
| Under certain conditions, n (%) | 67 (84.8) | 65 (80.2) |  |
| Never, n (%) | 0 (0) | 0 |  |

*Q8:* Do you switch anticoagulation from DOACs to VKAs in patients who suffer an AIS under DOAC treatment?

|  | **Regional SU** | **Trans-regional SU** | p=0.39 |
| --- | --- | --- | --- |
| Always, n (%) | 1 (1.3) | 0 (0) |  |
| Under certain conditions, n (%) | 40 (50.6) | 36 (44.4) |  |
| Never, n (%) | 38 (48.1) | 45 (55.6) |  |

*Q10:* Do you switch anticoagulation from one DOAC to another DOAC in patients who suffer an AIS under DOAC treatment?

|  | **Regional SU** | **Trans-regional SU** | p=0.87 |
| --- | --- | --- | --- |
| Always, n (%) | 1 (1.3) | 0 (0) |  |
| Under certain conditions, n (%) | 73 (92.4) | 76 (93.8) |  |
| Never, n (%) | 5 (6.3) | 5 (6.2) |  |
